# Study on the Mobile Colistin Resistance (mcr-1) Gene in Gram-Negative Bacilli in a Rural Tertiary Care Hospital in Western Maharashtra

**DOI:** 10.7759/cureus.75569

**Published:** 2024-12-11

**Authors:** Kajal S Yadav, Satyajeet Pawar, Kailas Datkhile, Satish R Patil

**Affiliations:** 1 Department of Microbiology, Krishna Institute of Medical Sciences, Krishna Vishwa Vidyapeeth, Karad, IND; 2 Krishna Institute of Allied Sciences, Krishna Vishwa Vidyapeeth, Karad, IND

**Keywords:** colistin resistance, conventional pcr, gram negative bacteria, mcr-1 gene, multidrug resistant (mdr)

## Abstract

Background: Colistin, a last-resort antibiotic for treating multidrug-resistant Gram-negative bacterial infections, has increased resistance as a result of the emergence of the *mcr-1* gene. The *mcr-*1gene, which confers colistin resistance, is often carried on plasmids, facilitating its spread by horizontal gene transfer among bacterial populations. The rising prevalence of *mcr-*1*-*mediated resistance poses significant challenges for infection control and treatment efficacy. This study aimed to detect and investigate the prevalence of the* mcr-*1 gene among Gram-negative bacilli isolated from clinical specimens in a rural tertiary care hospital and to analyze the plasmid-mediated mechanisms of colistin resistance.

Materials and methods: A cross-sectional study was conducted over two years at Krishna Institute of Medical Sciences, Karad. Gram-negative bacilli were isolated from clinical specimens and identified using standard methodology. Antimicrobial susceptibility testing was performed by using the Vitek-2 Compact (bioMerieux, Marcy-l'Étoile, France) method and the colistin-resistance broth microdilution method (BMD). Polymerase chain reaction (PCR) was done for the presence of *mcr-*1 gene in colistin-resistant isolates.

Results: Out of 359 Gram-negative bacilli isolates, 93 (25.90%) demonstrated resistance to colistin. Among these resistant strains, the *mcr*-1 gene was identified in 13 (13.97%) of the isolates. The gene was predominantly found in *Pseudomonas aeruginosa* (8, 61.53%), followed by *Klebsiella pneumoniae *(3, 23.07%)*, Acinetobacter baumannii *(2, 15.38%) among the 13 isolates. Out of the various specimens received, *mcr-*1 gene was found in endotracheal tube (4, 30.76%), urine (4, 30.76%), pus (3, 23.07%), sputum (1, 7.69%), and blood (1, 7.69%). Colistin minimum inhibitory concentration (MIC) value for these resistant isolates ranged from 4 to 16 µg/ml.

Conclusion: The study highlights a significant prevalence of *mcr-*1 plasmid-mediated colistin resistance gene among Gram-negative bacilli in the hospital. This possibly highlights the frequent misuse of colistin in animal husbandry from this rural area. The findings underscore the importance of monitoring resistance patterns and implementing stringent infection control measures.

## Introduction

Colistin was first discovered in 1947 and has been extensively used in both clinical and aquaculture applications since the 1950s. However, its therapeutic use has been limited due to concerns over nephrotoxicity and neurotoxicity and, as well as the development of new broad-spectrum antibiotics. In recent decades, colistin has regained importance as a last-resort treatment for infections due to the rise of multi-drug-resistant bacteria, including *Acinetobacter baumanni, Pseudomonas aeruginosa*, and carbapenem-resistant Enterobacteriaceae (CRE) [1]. Two commercial versions of colistin, an antibiotic belonging to the polymyxin group, are available. In some countries, colistin sulfate is used topically and for animal production, whereas colistin methanesulfonate is utilized parenterally [2]. Another kind of polymyxin that is used in clinical settings is polymyxin B, which is given in its active form. Although it was initially employed in both human and veterinary medicine in 1950, *Acinetobacter baumannii*, *Pseudomonas aeruginosa*, and Enterobacterales that produce carbapenemase are among the multidrug-resistant Gram-negative bacilli that have been using colistin as a last resort in human infections in recent years [3]. The global emergence of colistin-resistant bacteria can be attributed to the overuse and improper use of colistin in both human and animal medicine. The development of colistin-resistant bacteria, however, can also occur without any previous colistin exposure, leaving medical practitioners to treat patients on their own [4]. Following the discovery of plasmid-mediated colistin resistance caused by the *mcr*-1 gene in late 2015, it is concerning that the prevalence of colistin resistance has grown to be a major worry [5,6]. The recently discovered mcr-1 gene encodes the enzyme phosphoethanolamine transferase, which facilitates plasmid-mediated resistance to colistin [7]. This new mechanism of resistance is concerning since it could lead to pan-drug resistance in Enterobacteriaceae members [8]. Numerous *mcr*-1-bearing plasmids with intricate dissemination mechanisms have been found to have infected at least six Enterobacteriaceae species [9].

This study was done to know the prevalence of colistin-resistant organisms and *mcr*-1 gene in Gram-negative isolates, as colistin resistance is increasing among Gram-negative isolates from western Maharashtra.

## Materials and methods

Study design

This study was designed as a cross-sectional analysis to investigate the prevalence and distribution of *mcr-*1 gene in Gram-negative bacilli.

Study period 

The research was conducted over 25 months, from June 2022 to July 2024.

Sample size

Sample size was calculated for maximum value using the formula

$n=\frac{\left( Z^{2}\times{p}\times{q} \right)}{L^{2}}$

Where L = precision = 5%, Z= confidence level of colistin resistance, i.e., for 95% confidence level Z = 1.96 ≅ 2, P = prevalence of colistin-resistant organism, q=100-p. According to a study by Poonam AR et al., prevalence was 34% [10]. So, p = 34, q = 66. With the above formula and values, the sample size was 359. 

Therefore, all consecutive specimens were studied until a total of 359 Gram-negative bacilli isolates were obtained and analyzed during the study period.

Data collection

Clinical specimens from Krishna Hospital and Medical Research Centre (KH & MRC) were collected and processed at the Department of Microbiology, Krishna Institute Medical Sciences, Karad.

Inclusion criteria

Non-repetitive, consecutive patients' clinical isolates of *Pseudomonas* spp*.*, *Acinetobacter *spp*.*, *E. coli,* and *Klebsiella *spp. were included in the study.

Exclusion criteria

Among Gram-negative bacilli, the ones that are naturally colistin-resistant (Ex. *Proteus, Serratia marcescens, Providencia, Morganella morganii, Vibrio cholera, and Brucella* spp*.*) were excluded from the study.

After Institutional Ethics Committee approval (Ref. No. KIMSDU/IEC/06/2022), clinical specimens from indoor patients (IPD) were collected for two years for culture and detection of colistin resistance from Gram-negative microorganisms as per standard methodology [11]. Informed consent was taken first from a patient. The Gram-negative bacteria were isolated from clinical specimens such as endotracheal secretion, pus, sputum, pleural fluid, urine, stool, cerebrospinal fluid, blood, and body fluids like ascitic fluid and other specimens like catheter tips and various prosthetic devices, etc. Processing of the specimen was done on blood agar, chocolate agar, and MacConkey’s agar [12]. VITEK 2 Compact (bioMerieux, Marcy-l'Étoile, France) identified the organism by using a Gram-negative ID card 21341 and an antibacterial susceptibility tested by using AST-N405 and AST-N406 [12].

Phenotypic detection by broth microdilution method

The broth microdilution (BMD) method was performed as per Clinical & Laboratory Standards Institute (CLSI) standards, with a 96-well microtiter plate (HiMedia, Thane, India), colistin sulfate powder (Sigma Aldrich, St. Louis, MO USA), and cation-adjusted Mueller-Hinton broth (CAMHB). *Pseudomonas aeruginosa* American Type Culture Collection (ATCC) 27853 was used as the negative control, while *Escherichia coli* National Collection of Type Cultures (NCTC) 13846 (mcr-1-positive) was used as the positive control [10].

The first colistin sulfate stock solution was prepared by using sterile distilled water, sealed in sterile plastic vials, and kept at -70 °C until needed. In accordance with CLSI, a working stock solution was prepared from this stock solution by double dilution at concentrations ranging from 16µg/ml to 0.5µg/ml. The minimum inhibitory concentration (MIC) was identified as the lowest colistin sulphate concentration at which no discernible growth was seen. It was done to manage CAMHB's sterility, growth, and ATCC. Colistin sulphate minimum inhibitory concentration ≤ 2µg/ml was considered the threshold for susceptibility in the cases of *Pseudomonas aeruginosa*, whereas colistin sulphate MIC ≥4 was considered the threshold for resistance *Pseudomonas *spp., *Acinetobacter *spp., *E. coli*, and *Klebsiella *spp. [10].

Genotypic detection

Polymerase chain reaction (PCR) study was done for the prevalence of the plasmid-mediated* mcr*-1 gene in 93 colistin-resistance isolates. Plasmid DNA extraction was performed by HiPurA® Plasmid DNA Miniprep Purification Kit (HiMedia).

In Vitro Procedure of PCR for Genotypic Study

The colistin resistance gene was detected genotypically using the gold standard PCR method. Plasmid DNA was extracted from the colistin-resistant isolates using a plasmid DNA extraction kit, following the manufacturer’s protocol. Plasmid DNA from overnight-grow in cultures of selected isolates was added in 25 ml of the tryptose phosphate broth liquid medium and incubated at 37±2˚C in the incubator. Plasmid DNA concentrations were measured using a spectrophotometer (Shimadzu, Kyoto, Japan) by assessing absorbance at 260/280 nm, and the quality of the plasmid DNA was evaluated on 1% agarose gel electrophoresis after staining with ethidium bromide.

Confirmation of colistin resistance genes *mcr*-1 was detected by using PCR reactions. The PCR was initially optimized to generate all potential amplicons. The extracted plasmid DNA of each isolate was subjected to a polymerase chain reaction for amplification of the colistin resistance gene *mcr*-1. PCR amplification was performed in a 20 μL reaction mixture containing 1X PCR buffer (10 mM Tris-HCl, pH 8.3, 1.5 mM MgCl₂, 50 mM KCl), 200 μM of each dNTP's, 1 U of Taq DNA polymerase enzyme (Merck Millipore, Burlington, MA, USA), and 0.2 nM 309 bp of *mcr*-1 primer. Subsequently, 200 nanograms (ng) of purified plasmid DNA from each sample were added to the reaction mixture. The PCR amplification was performed using a Master Cycler gradient PCR machine (Eppendorf, Hamburg, Germany). Table 1 describes the PCR amplification that was carried out according to the following program: Initial denaturation at 95ºC for seven minutes, followed by 35 cycles of one-minute denaturation at 95ºC, one-minute annealing at 65ºC, and one-minute extension at 72ºC, finished with a final extension at 72ºC for 10 minutes. Following amplification, the products were analyzed by 2.0% agarose gel electrophoresis in 1X TAE buffer. Ethidium bromide (10 mg/ml) was used to stain the gel, which was then visualized under UV light and photographed using a gel documentation system (Bio-Rad Laboratories, Hercules, CA, USA). All the extracted isolates were run along with a 1500-bp DNA ladder, a molecular weight marker for confirmation of the specific size of the corresponding gene fragment. Also, positive control strains *Escherichia coli* NCTC13846 (*mcr*-1 positive) were used as standards for the presence of the *mcr*-1 gene.

**Table 1:** Details of the primers and thermal program used for the amplification of mcr-1 genes. PCR: Polymerase Chain Reaction, mins: minutes

Sr. No	Gene	Primer sequence (5'-3')	Product Size	Reaction conditions		
				PCR Steps	Temp.& Time	Cycles
1)	*mcr*-1	(5'-CGGTCAGTCCGTTTGTTC-3') (5'-CTTGGTCGGTCTGTAGGG-3')	309bp [13]	Initial denaturation	95°C- 7mins	35
				Denaturation	95°C-1mins	
				Annealing	65°C-1mins	
				Extension	72°C-1mins	
				Final Extension	72°C-10mins	
				Holding	4°C-∞	

## Results

**Figure 1:**
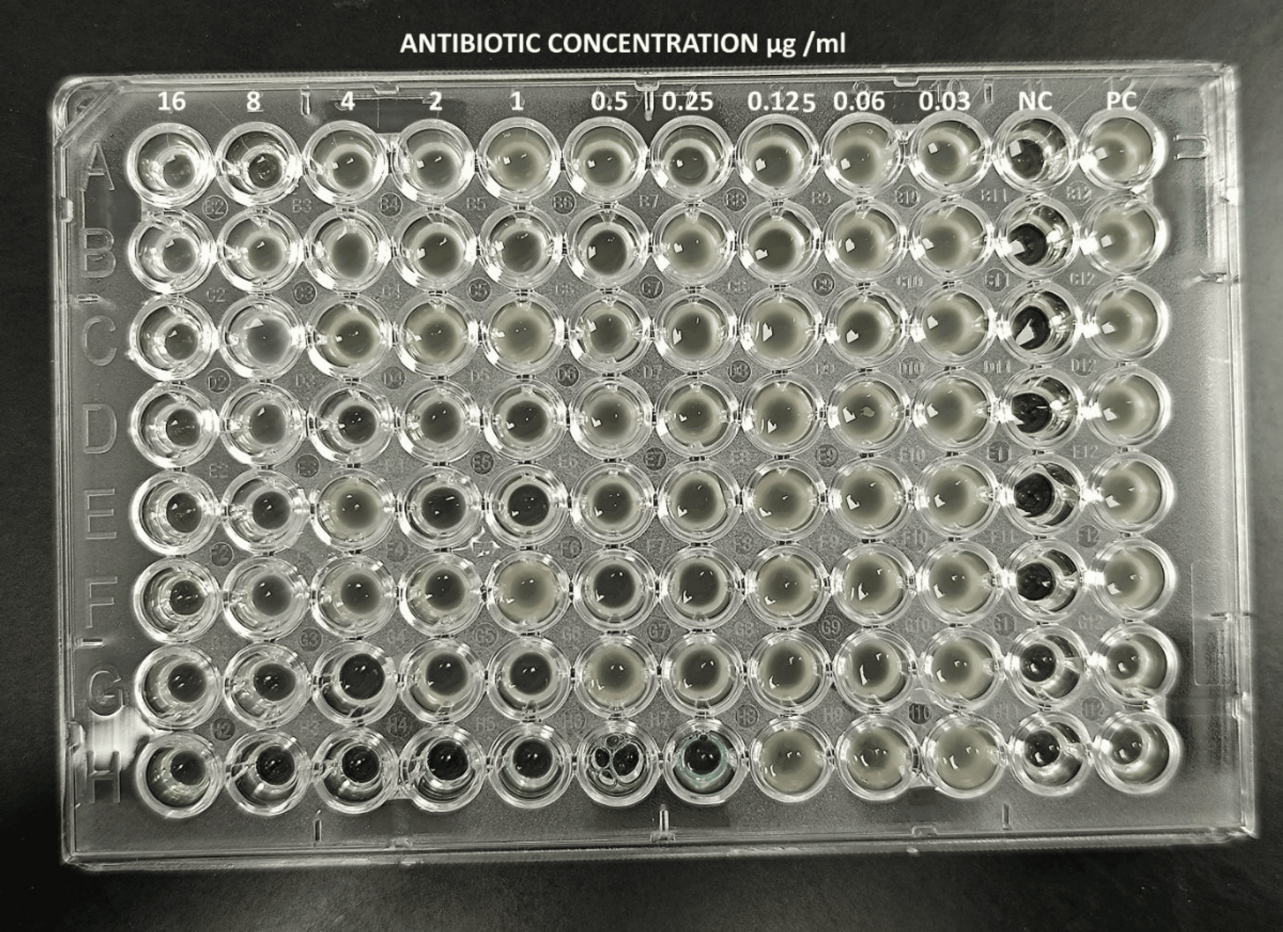
96-well microtiter plates containing Pseudomonas aeruginosa American Type Culture Collection (ATCC) 27853, used as a negative control, and Escherichia coli National Collection of Type Cultures (NCTC) 13846, used as a positive control, along with other tested organisms. NC: Negative Control, PC: Positive Control

In this study, out of 359 consecutive non-repetitive Gram-negative bacilli isolates, 93 (25.90%) demonstrated resistance to colistin by BMD. Among these resistant strains, the *mcr*-1 gene was identified in 13 (13.97%) of the isolates by PCR method. A total of 359 isolates were tested using VITEK 2 Compact and broth microdilution method, and the results were analysed for detection of the prevalence of colistin resistance isolates. Among 359 isolates tested by VITEK 2 Compact, 291 (81.05%) isolates were sensitive, and 68 (18.94%) were found to be resistant by VITEK 2 Compact. Figure 1 shows 359 Gram-negative organisms tested by BMD, 266 isolates (74.09%) were found to be susceptible to colistin sulphate (MIC ≤ 0.5 µg/ml), and the rest (93, 25.90%) isolates were resistant to colistin.

**Figure 2:**
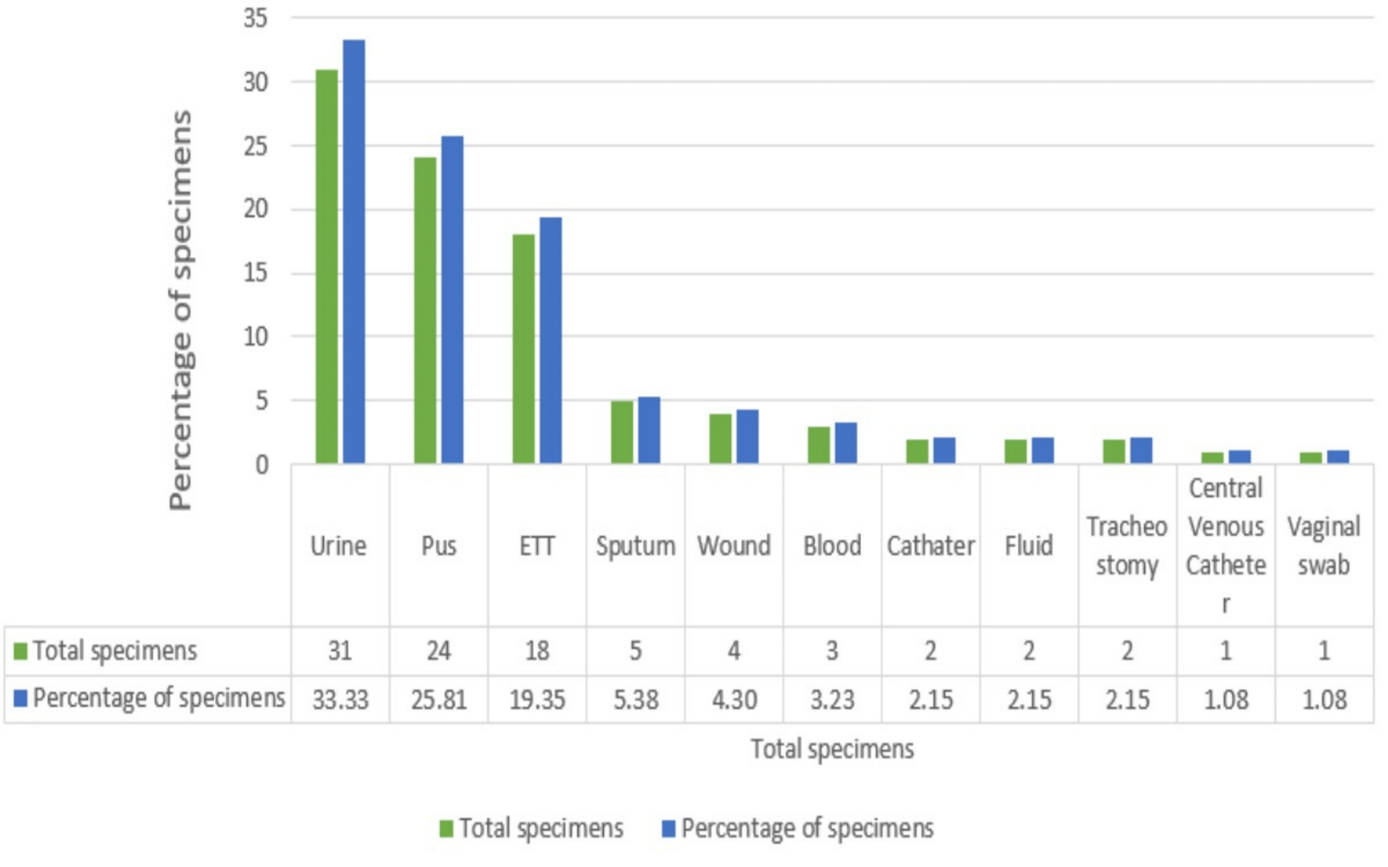
Distribution of colistin resistant isolates among clinical specimens. ETT: Endotracheal Tube.

In this study, as shown in Figure 2, out of the various specimens received, the highest number of colistin-resistant isolates were found in urine (31, 33.33%), followed by pus (24, 25.81%), endotracheal tube (18, 19.35%), sputum (5, 5.38%), wound (4, 4.30%), blood (3, 3.23%), catheter (2, 2.15%), fluid (2, 2.15%), tracheostomy (2, 2.15%), central venous catheter (1, 1.08%), and vaginal swab (1, 1.08%). 

**Figure 3:**
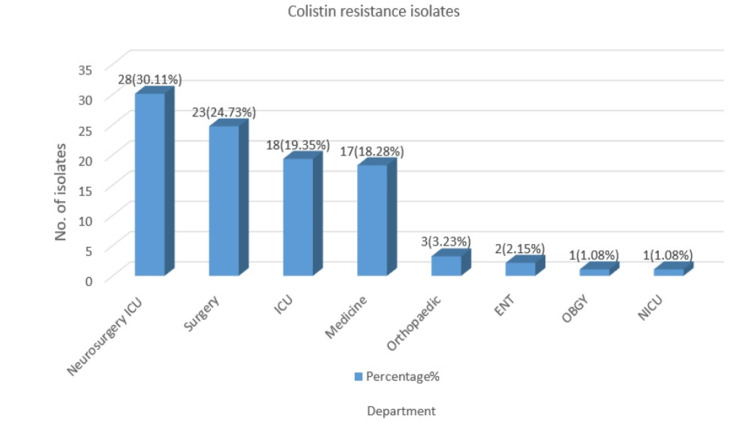
Ward-wise distribution of colistin-resistant isolates ICU: Intensive Care Unit, ENT: Ear, Nose, and Throat, OBGY: Obstetrics and Gynecology, NICU: Neonatal Intensive Care Unit.

Intensive Care Units (ICUs) are the primary source of multidrug resistance in hospitals. As shown in Figure 3, the majority (28, 30.11%) of colistin-resistant isolates were from the Neurosurgery ICU (28, 30.11%) followed by a surgery ward (23, 24.73%), ICU (18, 19.35), and medicine (17, 18.28%), orthopaedics (3, 3.23%), ENT (2, 2.15%), Obstetrics and Gynecology (1, 1.08%), NICU (1, 1.08). 

**Figure 4:**
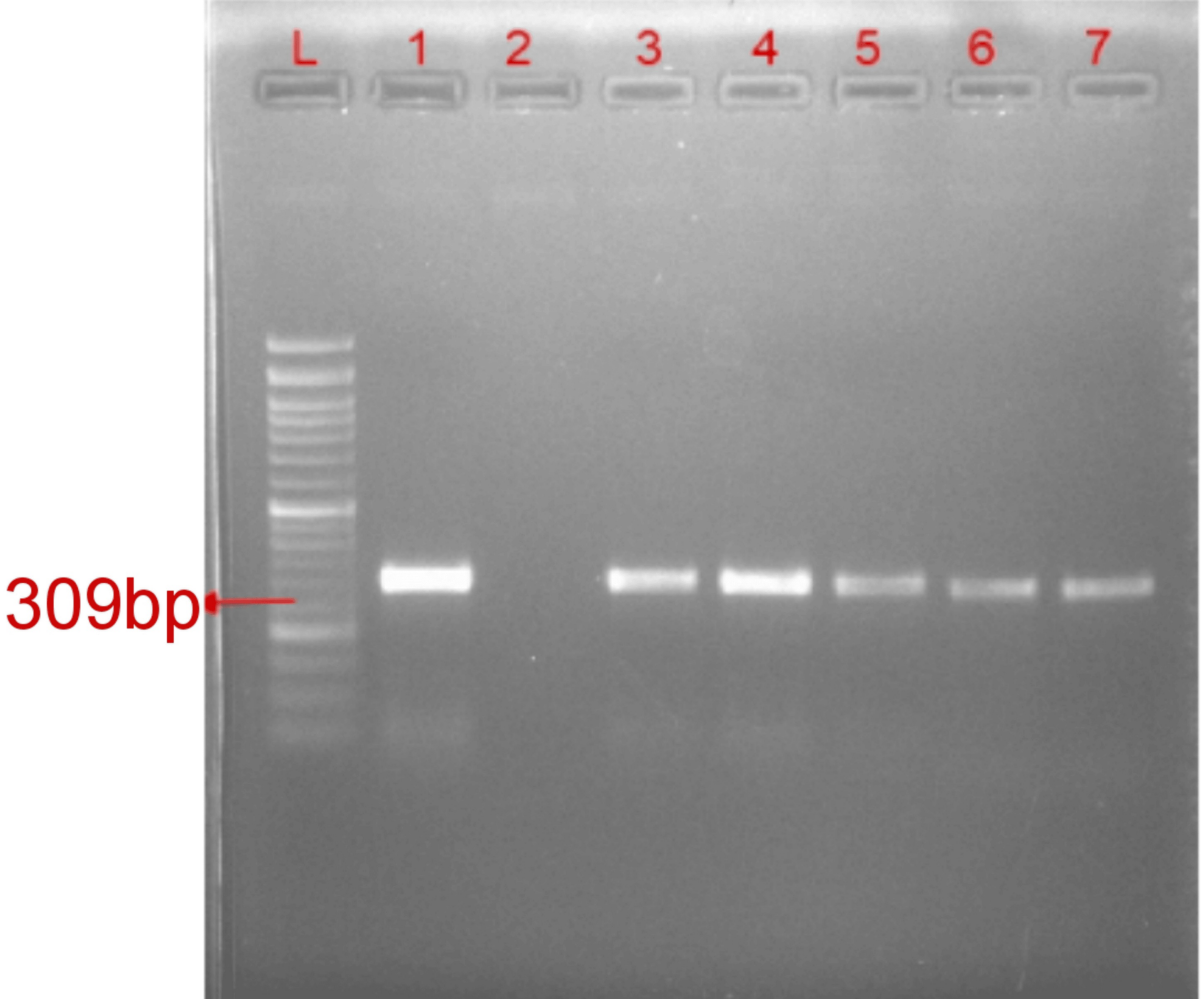
Amplification of mcr-1 genes Lane L was loaded with the 50 base pair (bp) DNA ladder. Lane 1 is a positive control for mcr-1 (309bp), Lane 2 is a negative control, and Lanes 3-7 are mcr-1-positive isolates for mcr-1.

As shown in Figure 4, amplification of the *mcr*-1 gene having a band size of 309 bp was observed on the agarose gel electrophoresis. In this present study, the gene was predominantly found in *Pseudomonas aeruginosa* (8, 61.53%), followed by *Klebsiella pneumoniae* (3, 23.07%) and *Acinetobacter baumannii* (2, 15.38%) among 13 isolates. Out of the various specimens received, the *mcr*-1 gene was prominently found in ETT (n = 4), followed by urine (n = 4), pus (n = 3), blood (n = 1), and sputum (n = 1). Colistin resistance organisms were seen in 93 (25.90%) isolates by the phenotypic method, and a genotypic method for *mcr*-1 showed positive results in 13 (13.97%) isolates. The colistin MIC value for these resistant isolates ranged from 4 to 16 µg/ml.

## Discussion

Indiscriminate antibiotic use has resulted in the growth of colistin-resistant Gram-negative organisms in critical care unit patients. As per result, susceptibility testing procedures are increasingly needed [12].

In the present study, colistin-resistant organisms were most frequently found in urine samples (33.33%), followed by pus (25.81%), endotracheal tube (19.36%), sputum (5.38%), wound swabs (4.30%), blood (3.23%), catheters (2.15%), fluid samples (2.15%), tracheostomy sites (2.15%), central venous catheter (CVC) line samples (1.08%), and vaginal swabs (1.08%). These findings are consistent with the study by Arjun et al. [14], which also identified urine samples (33%) as the primary source of colistin-resistant isolates, followed by respiratory samples (20.8%), pus (16.67%), blood (6%), and cerebrospinal fluid (4.17%). The study of Zaki et al. [15] reported the highest proportion of colistin-resistant isolates in urine samples (46%), followed by blood samples (30%) and exudates (24%), showing a broadly similar distribution pattern among the samples [14,15].

The discovery by Liu et al. (2015) that the colistin resistance *mcr*-1 gene was horizontally transferred across bacterial strains via plasmids raised concerns about the use of colistin as a last line for treating Gram-negative bacterial infections [5]. 

This study's colistin-resistant Gram-negative isolate prevalence was similar to that of Walkty et al., who discovered a 0.04% prevalence of the *mcr*-1 gene in colistin-resistant isolates [16]. A recent research investigation from Jordan found that patients with urinary tract infections were the first to have colistin-resistant *E. coli *that had *mcr*-1 [17]. This demonstrates the potentially harmful health consequences associated with plasmid-carried colistin-resistant genes in *Escherichia coli* [17]. According to a thorough analysis of hundreds of English and Chinese publications studied by Elbediwi et al., the *mcr* gene was found in 47 nations and areas on six continents, with the *mcr*-1 variant being responsible for 95% prevalence in 2018 [18]. *Acinetobacter baumannii *was shown to have a high prevalence of colistin resistance in an Iraqi investigation, with the *mcr*-1 gene found in 73.5% of the 121 strains taken from different hospitals in Baghdad [19]. Currently, the proportion of human-associated *mcr*-1 genes reported in various nations is much lower than that of animals, which may be closely related to colistin's restricted therapeutic use [20]. Bernasconi et al. identified the mcr-1 gene in four out of 38 tourists (10.5%) returning from India, with the exception of one individual who already carried the mcr-1 gene before the trip [21]. As reported by the "One Health" paradigm, the environment plays a crucial role in antibiotic resistance administration, and aquatic settings are regarded to be a perfect breeding ground for the spread of the *mcr*-1 gene [22]. The *mcr*-1-positive bacteria were discovered in numerous specimens taken from urban, community, rural, and natural habitats [23]. The establishment of plasmid-mediated colistin resistance in Gram-negative bacilli is currently a critical topic because of the significant risk of its spread in clinical settings. It is necessary to set appropriate rules against using this last-line therapy option in order to limit the increase of colistin resistance to gain a better knowledge of the present scenario of worldwide colistin resistance, clinical microbiology laboratories should develop and apply quick protocols for detecting colistin resistance [24].

Limitation

The study has few limitations in its genetic and microbiological approaches. It primarily focuses on detecting the *mcr*-1 gene while excluding other colistin resistance genes, such as *mcr-*2 and *mcr*-3, which may also play a role in resistance mechanisms. Furthermore, the study does not examine chromosomal mutations that could contribute to colistin resistance, limiting the understanding of other potential genetic factors involved. These limitations highlight the need for more comprehensive investigations to fully capture colistin resistance’s complexity.

## Conclusions

The study reveals a high prevalence of the *mcr-*1 plasmid-mediated colistin resistance gene among Gram-negative bacilli in the hospital, posing a serious public health concern. Given that colistin is a last-resort treatment for multidrug-resistant (MDR) infections, this finding suggests potential overuse in rural animal husbandry. The results highlight the urgent need for monitoring antimicrobial resistance patterns, implementing stringent infection control measures, and promoting judicious antibiotic use to mitigate the spread of resistance and safeguard critical therapies.
